# Comparing predictions of anger in conflict situations: Recalibrational Theory versus Dark Triad traits

**DOI:** 10.1017/ehs.2025.10030

**Published:** 2025-12-23

**Authors:** Isabella Righi, Mauro Silva Júnior

**Affiliations:** Department of Basic Psychological Processes, University of Brasília (UnB), Brasília, Brazil

**Keywords:** replication, evolved psychological mechanisms, welfare trade-off ratio, anger, Dark Triad Personality

## Abstract

Two research branches in evolutionary psychology can make similar predictions about treatment expectations in contexts of conflict of interest, where, for those involved, costs and benefits are at stake. Recalibrational Theory of Anger suggests that evolved psychological mechanisms operate at the cognitive level and regulate human behaviour. The Dark Triad Personality posits that traits of Machiavellianism, Narcissism, and Psychopathy confer adaptive advantages, leading individuals to prioritize their interests over those of others. This study aimed to conduct a direct replication of a previously experimental study on anger in conflict-of-interest situations in a Brazilian sample (Replication Analysis) and investigated whether dark triad traits predict the magnitude of anger in conflict-of-interest situations (Extension Analysis). The Replication Analysis consistently replicated previous findings, with effect sizes from moderate to large magnitudes. The Extension Analysis revealed that only Narcissism was a significant predictor when victims were intentionally targeted by offenders. While the Recalibrational Theory of Anger predictions were largely confirmed, the dark triad personality traits, except for Narcissism, were generally poor predictors of anger magnitude. The results suggest that the universality of the information processing is robust and is little influenced by antisocial personality characteristics.

## Graphical abstract

**Figure:**
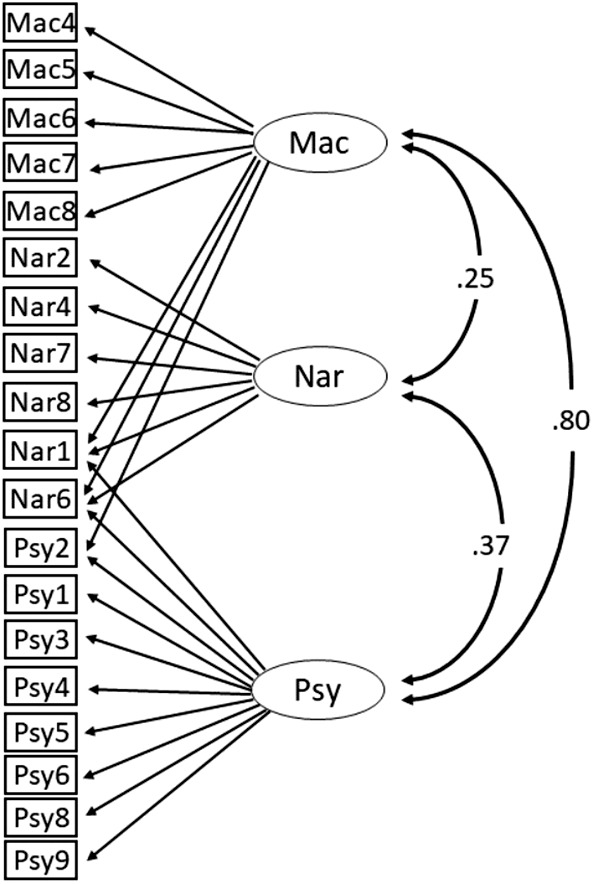

## Social media summary

The anger system’s information-processing architecture is robust and minimally affected by dark personality traits.

## Introduction

Evolved psychological mechanisms (EPMs) are fast and frugal solutions to adaptive problems that were selected in the ancestral past because they supposedly impacted the fitness of our ancestors (Silva Júnior, 2023; Tooby & Cosmides, 2015). EPMs are flexible and regulatory, functionally contingent on environmental information, activated by specific environmental stimuli (inputs), and produce adaptive behaviours in response (outputs). They constitute adaptations shaped by a history of selection and operate in diverse environmental circumstances, even those not experienced by our ancestors (Tooby & Cosmides, 2015). In cases of conflict of interest, for example, when an individual is not valued by another as expected, the inferior treatment is the recurring condition in which EPMs operate, promoting adaptive behaviours aimed at negotiating for more favourable treatment, based on contextual cues such as perceived costs and benefits (Sell et al., 2017; Tooby & Cosmides, 2008).

## Social emotions

Social emotions can be understood as psychological adaptations that were naturally selected in complex and conflicting social situations, related to decision-making processes. Social emotions, such as anger, perform crucial evolutionary functions for human sociability, activating EPMs to solve adaptive problems that involve processes of collaboration and social exchanges (Sznycer et al., 2021).

Its adaptive functions were designed to guide behaviours considering costs and benefits, trade-offs, for the ‘self’ and the ‘other’ (Sznycer et al., 2017, 2022). Anger encourages the ‘other’ to value more the well-being of the ‘self’; gratitude strengthens cooperative relationships; while shame reduces the dissemination of harmful information about oneself, seeking to inhibit devaluation (Sznycer et al., 2021).

## Regulatory internal variables and welfare trade-off ratio

Evolutionary psychologists hypothesize that one of the recurring adaptive problems is responding to socially devaluing situations, in other words, not being treated as expected (Sell et al., 2017). Valuing the well-being of the ‘other’ involves costs and benefits that depend on the ‘social value’ that the ‘other’ has for the ‘self’ and contextual conditions (Sznycer & Lukaszewski, 2019). Faced with the recurring adaptive problem of human beings making decisions that consider the well-being of others, natural selection may have shaped adaptations responsible for regulating and calibrating the limits of these interactions, so that the ‘self’ seeks to recalibrate in the ‘other’ how much it values their well-being (Tooby & Cosmides, 2008). Social emotions may have evolved as they offered reliable solutions to this dilemma (Sznycer et al., 2021). The human mind stores relevant information about people and situations that influence decision-making and regulate behaviour through internal regulatory variables (Tooby & Cosmides, 2008). The integration of this information configures a hypothesized welfare trade-off ratio (WTR), a variable used to calculate how much an individual weighs the well-being of another in relation to their own decision-making processes (Delton & Robertson, 2016).

WTRs help to understand the acceptable limits of the cost–benefit relationship in the relationship between individuals (Sell, 2017). By hypothesis, an actor’s WTR towards a target inclines the actor to provide help when WTR × *b* > *c*, where *b* = benefits given to the ‘other’ and *c* = costs incurred by the ‘self’ to provide benefits to the ‘other’. High WTRs promote benefits towards the ‘other’, while low WTRs are characteristic of relations in which one favours one’s own well-being (Delton & Robertson, 2016). WTRs are specific to each situation, and involve motivational, emotional, and cognitive processes; they regulate social relationships based on respect, reciprocity, love, and friendship (Sell, 2017). Evolutionarily, its function might be to regulate cost–benefit transactions – trade-offs – between one individual and another, in a way that favours fitness (Sell et al., 2017).

## Anger

People have intuitive knowledge about the triggers of anger, often translated into clues that an individual’s beliefs and intentions about another are incongruent with the expected levels of respect and importance (Sznycer et al., 2022). Functionally, anger has a communicative nature, seeking to obtain fairer treatment (Averill, 1982; Sznycer & Lukaszewski, 2019). The anger system may have evolved as a neurocognitive programme to deal with the adaptive problem of being insufficiently valued when greater recognition was expected since social evaluation can influence social exchanges (Sznycer et al., 2022). Lack of appreciation can result in the imposition of costs or withholding of benefits, while appreciation promotes help, support, and donation of benefits. The processing of anger-related information aims to adjust imbalances in this system, promoting fitness by enabling individuals to negotiate for more favourable treatment, especially when they possess greater bargaining power (Sell et al., 2017).

Anger is an important social emotion, recognized as universal and transcultural, with early ontogenetic development and signalled by widely recognized facial expressions and physiological responses (Ekman, 1999). It is marked by expressive universal signs, such as furrowed eyebrows, narrowed eyes, and pursed lips, which serve not only to communicate a willingness to confront but also to exaggerate cues of physical formidability – similar to how a cat erects its fur to appear larger during confrontations (Sell, Cosmides & Tooby, 2014). Physiologically, anger activates the sympathetic nervous system, resulting in increased heart rate, elevated blood pressure, and increased muscle tension (Ekman, 1999; Levenson, 1994). This profile suggests that anger is adaptively designed to prepare the organism to face conflict or threat situations, while simultaneously signalling to others the need to readjust behaviours or social relationships (Sznycer et al., 2022).

Although commonly associated with aggression, anger has an essentially communicative function, seeking to adjust the behaviour of others, rather than causing severe harm (Averill, 1982). Its expression demonstrates strength and willingness to escalate the conflict if necessary (Sznycer et al., 2022). Angry individuals confront the offender, questioning their motivations, and escalation of aggression occurs if the target does not adopt a conciliatory stance (Felson, 1982). An effective apology signals an internal change in behaviour or intention and is effective in defusing anger (Frantz & Bennigson, 2005).

The Recalibrational Theory of Anger proposes that anger acts as an evolutionary bargaining strategy to resolve conflicts of interest in favour of the angry individual (Sell, 2017; Sell et al., 2009). Bargaining for better treatment aims to recalibrate the other’s WTR, encouraging the ‘other’ to adjust their behaviour to value the well-being of the ‘self’ in decisions involving costs and benefits (Sell, 2017). Anger is triggered when perceived WTR is lower than expected, responding to specific environmental cues. Its intensity varies according to the magnitude of the cost imposed, the magnitude of the benefit obtained by the ‘other’ and the intentionality in the action of inflicting a cost (Berkowitz & Harmon-Jones, 2004; Sell et al., 2017).

## Cross-cultural evidence

As universal adaptations, EPMs are observed across diverse cultures (Lordelo, 2010; Silva Júnior, 2023). Sell et al. (2017) investigated the universality of anger processing through experiments conducted in five industrialized societies (USA, Australia, Romania, India, Turkey) and one non-industrialized group (the Shuar people of Ecuador). Their results demonstrated that anger-related information processing is specifically activated in response to manipulations of costs and benefits during social conflicts, aiming to recalibrate the WTR when an individual perceives themselves as undervalued. Additionally, individuals may feel anger for numerous reasons, such as insults, insufficient reciprocity, neglect, etc. (Sell, 2011).

Interestingly, greater inequity – when the offender enjoys a large benefit – was associated with less anger, contradicting predictions from Equity Theory, which anticipates a positive relationship between anger and the benefits others receive. These findings also challenge social constructivist models that emphasize cultural variation in anger processing. Instead, Sell et al.’s (2017) results support the universality of anger’s cognitive-emotional architecture, which functions consistently across cultures.

## Individual differences and the Dark Triad

Although EPMs are supposed to have a functional design shared by all members of the species, individual differences and environmental cues can influence the magnitude and expression of emotions, both of which are outputs of EPMs (Sznycer & Lukaszewski, 2019). Therefore, Personality Psychology seeks to build a theory that integrates universal aspects and individual differences, as both shape thoughts, emotions, and behaviours. The adaptationist personality framework explores intrapersonal, interpersonal, and cross-cultural variations generated by EPMs and how these individual differences impact fitness (Lukaszewski et al., 2020).

The ‘dark side’ of the human personality is characterized by traits with antisocial attributes, manifested in different contexts and intensities (Zeigler-Hill & Marcus, 2016). The Dark Triad Personality comprises three interrelated, aversive, and non-pathological domains (Narcissism, Machiavellianism, and Psychopathy: Paulhus & Williams, 2002) and describes the tendency to prioritize one’s own well-being over those of others. Individuals with high levels of these traits tend to adopt antisocial and self-promoting behaviours to achieve their goals (Lukaszewski et al., 2020).

The domains of Dark Triad share some core characteristics, such as manipulative behaviours, lack of empathy, self-promotion, and antisocial tendencies (Jones & Paulhus, 2014; Schreiber & Marcus, 2020), but they also have specific characteristics: Narcissism stands out for its high sense of grandiosity, extreme need for recognition and egocentrism. Meanwhile, Machiavellianism presents a characteristic tendency for manipulation, strategism, formation of alliances, and willingness to delay immediate gratification to achieve great rewards. Finally, Psychopathy is marked by hostility, impulsiveness, and insensitivity, in addition to the constant search for rewarding sensations (Jones & Paulhus, 2014; Koehn et al., 2019).

Evolutionary psychologists test hypotheses to evaluate the possible evolutionary benefits of social traits that are aversive to others, such as manipulativeness, callousness, and exploitation (Jones & Paulhus, 2010). According to this perspective, dark traits are strategies shaped by natural selection that aim to maximize personal gains in response to environmental conditions, such as resource scarcity, high competition, or unstable social environments (Jonason & Middleton, 2015). Resource allocation and adaptation strategies vary according to socio-ecological conditions, influencing the development of personality traits as adaptive responses to the environment (Barbosa & Silva Júnior, 2023; de Mello & Silva Júnior, 2023). The literature indicates that dark traits are more prevalent in unstable and resource-scarce contexts, where exploitative and competitive behaviours can be adaptively advantageous (Jonason & Middleton, 2015; (Barbosa & Silva Júnior, 2023; de Mello & Silva Júnior, 2023)).

## Experimental replication

In the context of evolutionary psychological theory and research, the advancement of scientific knowledge through replication plays a fundamental role. It allows validation of the universality of EPMs, generalizing hypotheses to different contexts and populations, and confirming their adaptive and transcultural character (Cosmides et al., 2010).

Disagreements regarding the importance of replication in psychological studies have been overcome by the growing recognition of its indispensability for scientific improvement and the continuous dialogue between theory and evidence (Nosek et al., 2021). Collective efforts such as the Reproducibility Project, which sought to replicate original psychological studies, revealed that only 36% of replicated studies produced statistically significant results similar to the originals, although 97% of the original studies were statistically significant (Open Science Collaboration, 2015).

Replication is essential for improving research practices and driving scientific progress, as non-replicable findings compromise prediction and theoretical development (Stevens, 2017). Without replication, there is a risk of basing knowledge on contextually specific or coinciding results, an inadequate basis for theoretical reasoning and foundations (Cohen, 1994). Replicable results allow for continuous reviews of their meaning and validity, promoting advances in knowledge. Thus, replicability acts as an effective tool in generating and testing hypotheses, challenging established understandings, and stimulating innovation (Nosek et al., 2021).

## Research question and objectives

From an evolutionary psychological perspective, the sense of entitlement, present in dimensions of antisocial personality, can impact the expression of anger (Sell et al., 2009). Given that individuals with high levels of Dark Triad traits tend to prioritize their own well-being, and anger seeks to recalibrate WTRs in relationships, it is reasonable to suspect that the magnitude of anger is expected to be greater in individuals with higher levels of Dark Triad traits.

Therefore, this study has two main objectives. Firstly, we aimed to reproduce (Replication Analysis) the study by Sell et al. (2017), and secondly, to verify whether the dark personality is a predictor of the change in magnitude of anger in the experimental conditions used in Sell et al.’ study (Extension Analysis).

## Hypotheses and predictions

Based on the theories described previously, we constructed a set of hypotheses and predictions concerning both analyses, which are described below:

### Replication Analysis

**Hypothesis 1:** The magnitude of anger varies depending on the cost and benefit transactions between offenders and the offended individual.

The WTR represents the degree to which an individual values another’s welfare relative to their own. A low WTR reflects a reduced willingness to maintain or restore cooperative relationships and typically occurs when the offender imposes high costs on the offended individual while obtaining only minor personal benefits. This situation signals that the offender’s WTR towards the victim is low, which in turn elicits anger in the offended individual as an adaptive response aimed at recalibrating social exchanges and encouraging the offender to show greater consideration and respect for the victim’s welfare. Therefore, the predicted variations in anger intensity across different cost–benefit scenarios can be understood as reflections of shifts in the WTR between individuals.

**Prediction 1:** Holding constant the benefit received by the offender, anger will become more intense as the cost imposed on the offended individual increases.

**Prediction 2:** Holding the imposed cost constant, anger will become less intense as the benefit received by the offender increases.

**Prediction 3:** Holding costs and benefits constant, anger will be more intense when the offender deliberately imposes the cost on the offended party rather than imposing the cost randomly.

**Hypothesis 2:** When confronted by an angry individual, upon whom he or she has imposed a cost, the offender should prefer arguments that indicate a high WTR relative to the angry individual.

**Prediction 4:** If the offender has a high WTR for the offended party, he should prefer to argue that the cost imposed was small.

**Prediction 5:** If the offender has a high WTR for the offended party, he should prefer to argue that the benefit received was large.

**Prediction 6:** If the offender has a high WTR for the offended, he should prefer to argue that he imposed a cost without knowing that he would deliberately inflict it on the offended.

It is important to clarify that these predictions are contingent on the offender possessing a sufficiently high WTR towards the offended individual. In other words, the offender feels ‘justified’ or ‘in the right’ to engage in argumentation aimed at minimizing the perceived harm caused. This high WTR reflects the offender’s motivation to maintain or restore a cooperative relationship by downplaying the cost imposed and emphasizing the benefits received. This framework aligns with the Recalibrational Theory of Anger, which focuses on the cost–benefit dynamics in social exchanges.

## Extension Analysis

Although the Dark Triad traits share characteristics such as egocentrism, manipulativeness, and callousness, each domain reflects distinct cognitive and motivational profiles that differentially modulate how individuals evaluate interpersonal costs and benefits. Machiavellianism is associated with a strategic cognitive style focused on maximizing instrumental gains through calculated and goal-directed social exchanges (Jones & Paulhus, 2014; Pilch et al., 2020). Accordingly, individuals high in Machiavellianism may perceive transgressions as more dysfunctional – and thus more costly – when the offender’s benefit is low, as such behaviour signals poor strategic value and imposes unnecessary interpersonal costs. In contrast, Narcissism, characterized by grandiosity, intense status-seeking, and a strong need for external validation (Miller et al., 2017; Zeigler-Hill & Marcus, 2016), may amplify the perceived costs of transgressions when the offender gains a high benefit, since this outcome represents a symbolic threat to the narcissist’s relative social value. In both cases, anger functions as a recalibration mechanism of WTRs, intensifying in response to perceived imbalance. Furthermore, whether the victim is specific or random may modulate this cost perception: Machiavellian individuals may respond more strongly to personal losses that directly affect their strategic interests, while narcissistic individuals may interpret arbitrary harm as a sign of disrespect towards their social position, regardless of who the immediate target is (Hyatt et al., 2018; Krizan & Johar, 2012). Based on this rationale, we originally set the following hypothesis and predictions.

**Hypothesis 3:** The Dark Triad Personality domains will be predictors of the magnitude of anger in situations of conflict of interest given the variation in costs and benefits for offenders and victims.

**Prediction 7:** Individuals scoring higher in Machiavellianism, compared to those scoring lower, will exhibit greater anger when the offender’s benefit is low.

**Prediction 8:** Individuals scoring higher in Narcissism, compared to those scoring lower, will exhibit greater anger when the offender’s benefit is high.

**Prediction 9:** Individuals scoring higher in Machiavellianism, compared to those scoring lower, will exhibit greater anger when the victim is specific.

**Prediction 10**: Individuals scoring higher in Narcissism, compared to those scoring lower, will exhibit greater anger when the victim is random.

During the review process, a reviewer noted that Predictions 8 and 10 were not perfectly aligned with the theories being tested (see Discussion), but we present them here for posterity as we originally proposed them.

## Method

### Participants

Two hundred and eighty-six people from Brazil participated in the research. After exclusions due to incomplete responses (61), 225 participants remained. Of these, 169 people identified themselves as female (75.1%), one person self-identified as intersexual (0.4%) and one person preferred not to answer about their sex (0.4%). The mean age of the participants was 33.5 years (SD = 14.53), ranging between 18 and 80 years. Being 18 years old or older was the inclusion criterion.

The sample was composed of participants with different levels of education, the majority of whom had completed postgraduate studies (33.3%), were studying higher education (31.6%), or had completed higher education (20.9%).

### Instruments

Data was collected and stored online via the SurveyMonkey platform, accessed through computers or smartphones. The online form consisted of a sociodemographic questionnaire, with questions about sex, age, and education, the experimental vignettes, scenarios translated and adapted from Sell et al. (2017), and the *Short Dark Triad Scale* (SD3), translated and adapted into Brazilian Portuguese (Monteiro, 2017). Instruments are available in the Supplementary Material (SM).

## Procedure

### Data collection

This study was conducted under the principles outlined in the Declaration of Helsinki and was approved by the Ethical Committee for Research in Human and Social Sciences of the University of University of Brasília under report number CAAE 75230423.6.0000.5540. Data collection occurred both online and in person, to verify whether the response environment influenced the participants’ performance. In the online modality, participants were invited through social media platforms such as WhatsApp and Instagram. In the in-person modality, data collection took place in the University of Brasília by using folders distributed and posted on bulletin boards, in addition to invitations made in the classrooms by the first author. A pilot study was carried out to evaluate experimental controls, and response time, and to seek refinements and improvements to the instrument (see SM).

## Experimental procedure

In both modalities, participants accessed the link to the form on the SurveyMonkey platform. Upon accessing, they read the invitation to participate (see SM), which briefly presented the research, including the estimated duration of 15 minutes for data collection. They then read the Informed Consent (see SM) and decided whether they would accept to participate, clicking the ‘I agree to participate’ button.

Participants were directed to the sociodemographic questionnaire and, subsequently, to the Short Dark Triad and vignettes sections. The order of presentation of the instruments was counterbalanced to control for order effects. After the sociodemographic questionnaire, participants randomly answered Short Dark Triad followed by the vignettes, or vice versa. Finally, they were directed to the acknowledgments section (see SM). The same participants completed both the experimental vignettes (Replication Analysis) and the Short Dark Triad scale (Extension Analysis; see details below). The Replication Analysis tested whether Sell et al.’s (2017) findings generalize to a different cultural context. The Extension Analysis examined whether Dark Triad traits predict the magnitude of anger observed in the replication.

## Experimental vignettes

The present study used three experimental vignettes (Telephone, Lunch, and Argument/Reaction) constructed by Sell et al. (2017). Just as in the case of the original study, which used translations and adaptations for different cultures, we translated and adapted the vignettes to the Brazilian context. The vignettes presented scenarios with social situations of conflict of interests, in which participants were invited to imagine how they would feel if they experienced the situations described. The objective of the experimental manipulation was not to induce anger, but to activate EPMs related to the judgment of social situations, in search of a cognitive assessment of the problem situation.

When reading the scenario described in Telephone vignette (Benefit conditions), participants were instructed to read a description and imagine themselves in a situation. In the situation they were in line at a public telephone to call a friend they will be late. They also had to make the call before their bus arrived, otherwise they would have to wait for an hour for another one. However, a person (the offender) cut in the line, making them miss the bus. The offender, in turn, calls someone and tries to avoid losing a lottery prize (Part 1). After reading this, participants were randomly assigned to one of two experimental conditions in which they received additional information that characterized the independent variable of the experimental manipulation: in the High Benefit condition, the offender avoided losing a large amount of money (R$5,000.00), while in the Low Benefit condition, they avoided losing a small amount (R$5.00) (Part 2).

In the Lunch vignette, participants read a scenario in which, during a university trip, a classmate puts a cockroach in their lunch while everyone is away. At lunch time, the students and the teacher come back and open their lunches. The participants open their lunch and find out a rude message and everyone sees the cockroach coming out of the lunch (Part 1). After reading this, participants were randomly assigned between two experimental conditions: in the specific victim condition, the offender knew who the lunch belonged to, while in the random victim condition, he did not know (Part 2). The participants did not take part in the same conditions. They were randomly assigned by the SurveyMonkey platform to one of the two Benefit conditions (high or low) and one of the two Victim conditions (specific or random).

In Part 1 of Telephone and Lunch vignettes, participants read the initial scenario and answered the item measuring the initial anger score, measured on a Likert scale from 1 (not at all *angered*) to 7 (extremely *angered*). In Part 2, they read the continuation of the scenario, answered an attention question, inserted to check their understanding of the initial scenario, and then the change in anger score measurement item, assessed on a scale from −3 (much less angry) to +3 (much angrier). Possible activation of six additional emotions (happiness, surprise, sadness, fear, envy, shame, and compassion), assessed in a 7-point Likert scale, were also evaluated in Part 2. In the in-person modality, an additional qualitative question asked participants to explain whether the intensity of anger had changed after the additional information and why. The objective here was to investigate implicit calculations of WTR. This qualitative data collection, however, only occurred in the in-person modality due to a methodological error.

In the Arguments/Reactions vignette, two scenarios were presented: that of argument scenario and that of reaction scenario, each accompanied by a list of six arguments that participants should evaluate. Participants were randomly assigned to respond to only one of the two scenarios. In the argument scenario, they were instructed to imagine that they had inflicted a cost on a social partner to obtain a personal benefit and to evaluate which arguments, in the role of the offender, could help reduce their friend’s anger. Example: the participant had ruined his friend’s shirt, and his friend would be very upset about not being able to wear it anymore, the participant should evaluate the arguments that would help him convince his friend that what he did was not so bad. This evaluation was done through a Likert scale, from −3 (does not help me) to +3 (definitely helps me) points. In the reaction scenario, participants had to imagine themselves in the opposite position, as victims, since they had suffered a cost in exchange for a benefit received by the partner. Example: the friend had ruined the participant’s shirt, and the participant would be very upset about not being able to wear it anymore. The participant should evaluate the arguments that would most or least irritate them by using a 7-point Likert scale – from −3 (definitely makes me less irritated) to +3 (definitely makes me angrier) – to indicate this. SM provides detailed descriptions of all the experimental vignettes and the attention questions used.

## Short Dark Triad

Developed by Jones and Paulhus (2014) to assess the three traits of the Dark Triad Personality (Narcissism, Machiavellianism, and Psychopathy), the Short Dark Triad (SD3) is a self-report scale consisting of 27 items, with nine items dedicated to each of the three domains. Participants responded to these items using a 5-point Likert scale (1 = strongly disagree; 5 = strongly agree). Examples of items include statements such as ‘I tend to manipulate others to get my way’ (Machiavellianism), ‘I like to be the centre of attention’ (Narcissism), and ‘I have used deceit or lied to get my way’ (Psychopathy). The measurement followed the procedure described in the original study (Jones & Paulhus, 2014), with the individual mean for each domain being calculated, as well as the overall sample mean for each trait.

## Power analysis

The G*Power software (version 3.1.9.7) was used to estimate, a priori, the sample size necessary for experimental replication in Replication Analysis. The calculation was based on the following parameters: expected moderate effect size (*d* = .5), the significance level of 5% (α = .05), and statistical power of 80% (*β* = .8). The choice of these parameters aimed to ensure that the study had sufficient power to detect significant differences between the groups. For the independent samples *t*-test, G*Power indicated the need for a sample of 128 participants (*N*1 = 67 and *N*2 = 69), with a critical value of *t* = 1.97. For the paired samples *t*-test, the software suggested a sample of 34 participants (*N*1 = *N*2).

To ensure that our study has sufficient statistical power to detect effects of interest, we conducted a sensitivity analysis using G*Power software (version 3.1.9.7). Specifically, we performed the analysis for a linear multiple model, considering the predictors, a power of .8, an alpha level of .05, our sample size, and three response variables (the SD3 domains). The sensitivity analysis indicated that the study has sufficient power to detect a minimum effect size of *f*^2^ = 0.04. This value is indicative of a small effect, according to Cohen’s (1988) benchmarks. Therefore, our study is adequately powered to detect the effects of the predictors, even if they are small.

## Statistical Analysis

### Replication Analysis

In Part 1 of Telephone and Lunch vignettes, the functionality of the stimulus was first verified, i.e., whether the described scenarios triggered the processing of information about the emotion of anger. The sample mean of the initial anger score was expected to be greater than or equal to 4 points (midpoint of the Likert scale), as in the studies by Sell et al. (2017). In Part 2, participants answered how much their perception of the magnitude of anger changed after inserting the independent variable of each experimental condition.

To verify whether the change in the anger magnitude was statistically significant between the experimental conditions (High Benefit *vs*. Low Benefit; Specific Victim *vs*. Random Victim), we performed independent samples *t*-tests and calculated effect sizes (Cohen’s *d*). The change in the magnitude was expected to differ significantly between conditions, presenting moderate to large effect sizes, as in the original study. Concerning the other six emotions investigated, the averages of the participants’ responses for each emotion were calculated and independent samples *t*-tests reported whether the emotions differed significantly between the experimental conditions (High Benefit *vs*. Low Benefit; Specific Victim *vs*. Random Victim). Details of these analyses may be found in the SM.

In the third vignette, for each scenario (argument and reaction scenarios), the arguments were compared according to their theoretical meaning concerning cost–benefit transactions. The means of the participants’ intra-subject evaluations were compared in the arguments that indicated low cost versus high cost; low benefit versus high benefit; specific victim versus random victim. The data were analysed using paired-sample *t*-tests that compared the average evaluation of each argument with its opposite and calculated the effect sizes (Cohen’s *d*). It was expected that, as in Sell et al. (2017), the preferred arguments were those that indicated high WTR of the offender over the victim and very high effect sizes, considering the intra-subject analysis. Cohen’s *d*s and False Discovery Rate correction for multiple comparisons were obtained using *R* programme 4.4.0.

## Extension Analysis

A set of confirmatory factor analyses (CFA’s) was carried out to evaluate the plausibility of a multidimensional structure for the SD3 scale. After adjusting the models based on the modification indices, Model 5 presented satisfactory fit indices. These indices were *χ*^2^ (*df*) = 258.826, *p* = .001, CFI = .91, TLI = .90, RMSEA = .060, SRMR = .074. The reliability measure used was composite reliability, which overcomes the problems found in more conventional measures such as Cronbach’s alpha and McDonald’s omega. The composite reliability of the Machiavellianism, Narcissism, and Psychopathy domains were 0.8, 0.63, and .87, respectively (see SM).

To verify whether Dark Triad Personality traits were predictors of the magnitude of anger, two multiple linear regression analyses were conducted. During the review process, one of the reviewers suggested performing a single multiple regression testing the interaction effects between the Dark Triad domains (Machiavellianism, Narcissism, and Psychopathy) and the experimental conditions (High benefit, Low benefit, Specific victim, and Random victim). However, we understood that at least two multiple regressions were necessary: one to predict the anger change score in the high versus low benefit conditions based on the three Dark Triad domains, and another to predict the anger change score in the random versus specific victim conditions based on the three domains.

To address this request, we first transformed the individual scores in each Dark Triad domain and the anger magnitude change score into *z*-scores (e.g., *ZMach* and *ZAngerTelephone*). Next, we mean-centred these values to reduce multicollinearity in the interaction terms of the regression (e.g., *Mach__centered_*). We created dummy variables from the experimental conditions, using Low Benefit and Random Victim as the reference categories.

From this point, we computed the interactions between the Dark Triad domains and the experimental conditions by multiplying the centred domain scores by the dummy variables, creating individual scores for each Dark Triad domain in each experimental condition (e.g., *Mach__benefit_* and *Mach__victim_*). The centred values were entered into the multiple regression as main effects, while the interaction scores were entered as interaction effects. The dependent variable was the anger change score. The Enter method was used for regression.

It was expected that the Dark Triad traits would modulate the magnitude of anger in situations of conflict of interest between social partners and there would be variation between the Dark Triad domains concerning its predictive effect, due to its central characteristics. In the low benefit and specific victim conditions, it was predicted that Machiavellianism would express a more significant predictive effect. Meanwhile, it was expected that Narcissism would present such a predictive effect in the high benefit and specific victim conditions.

## Results and discussion

### Results of Replication Analysis

There were no significant differences between the means of groups who participated online (*N* = 164) or in-person (*N* = 61) modalities. Similarly, we found no significant difference between the means of the groups who answered the attention question correctly and those who answered it incorrectly (see SM for details). Therefore, the results described in this study refer to data from all participants (*N* = 225).

## Telephone vignette

### Stimulus check: did the vignette trigger anger information processing?

The experimental vignette worked as expected: imagining the situation described (Part 1) should trigger the processing of anger information before participants knew why the subject cut in the line and took their place. As in the original study, in which the initial anger score averages of the cultures studied ranged from 5.1 to 6.3 points, the mean initial anger magnitude score in our study was 5.88 (SD = 1.41).

### Was the magnitude of anger greater when the subject suffered a cost for a small benefit rather than a large benefit?

In Part 2 of Telephone vignette, participants were randomly allocated to one of the experimental conditions, which manipulated the information about the reason for the imposed cost, 110 individuals from the High Benefit condition participated, with a mean of change in anger magnitude of −.86 (SD = 1.53). In the Low Benefit condition, the mean of change in anger magnitude was −.04 (SD = 1.45).

The *t*-test for independent samples revealed that, as in the study by Sell et al. (2017), the magnitude of change in anger was greater in the Low Benefit condition than in the High Benefit condition: *t*_(223)_ = −4.111, *p* = .001, *d* = −0.55, IC 95% [0.28, 0.81]. These results confirm Prediction 2 of Hypothesis 1 and indicate that by keeping the imposed cost constant, the magnitude of the victim’s anger decreases as the benefit received by the offender increases.

## Lunch vignette

### Stimulus check: did the vignette trigger anger information processing?

The functionality of the experimental vignette as an adequate stimulus to assess the magnitude of anger presupposes that imagining oneself in the situation described must trigger the processing of anger before participants know whether they were specific or random victims. As in the original study, in which the initial anger score averages of the cultures studied ranged from 4.5 to 5.7 points, the results show that the scenario worked as an appropriate stimulus to activate information processing, with our sample presenting *M* = 6.25 (SD = 1.17).

### Was the magnitude of anger greater when the subject knew that they were chosen as a specific victim instead of being a random victim of the prank?

In the specific victim condition, the mean of change in magnitude of anger was 1.67 (SD = 1.43). In the random victim condition, the mean of change in magnitude of anger was −.09 (SD = 1.13). Independent samples *t*-test showed that the magnitude of anger was significantly greater in the specific victim condition than in the random victim one: *t*_(223)_ = 10.315, *p* = .001, *d* = 1.37, IC 95% [1.08, 1.66]. These results confirm Prediction 3 and support Hypothesis 1, indicating that anger increases when the cost imposition is perceived as intentional rather than random.

## Argument/reaction vignette

Participants randomly responded to the argument scenario (*N* = 129) or the reaction scenario (*N* = 96). The list of six arguments presented in argument scenario and reaction scenario was divided, for data analysis, into contrasting pairs of costs and benefits (Pair 1: low cost *vs.* high cost; Pair 2: low benefit *vs.* high benefit; Pair 3: specific victim *vs.* random victim).

## Argument scenario

### What arguments do subjects prefer when confronted by an angry individual?

Participants were asked (i) to imagine that they had inflicted a cost on a friend to obtain a benefit for themselves and (ii) to evaluate arguments to convince the victim that their actions were not so serious. The average ratings of each argument with its opposite were compared to verify the preference for arguments that demonstrate high WTR from the participant to their offended friend.

The results reveal that participants preferred arguments indicating a high WTR towards the angry individual – specifically those emphasizing low cost (Argument 1), high benefit (Argument 4), and random victim scenarios (Argument 5) – and rejected arguments reflecting a low WTR – those emphasizing high cost (Argument 2), low benefit (Argument 3), and specific victim scenarios (Argument 6) – as shown in Table 1. The arguments were analysed considering their theoretical meaning, which was not presented to the participant (see details in the SM). The results confirm Predictions 4–6 investigated in this study and Sell et al. (2017).

**Table 1.** Anger magnitude by argument scenario

Group	Mean (SD)	*t* (*df*)	*P* adjusted	*d*	CI (95%)
Low cost	*M*_A1_ = − 0.48 (2.10)	9.59 (128)	.001	0.84	[1.71, 2.60]
High cost	*M*_A2_ = − 2.64 (1.22)				
Low benefit	*M*_A3_ = − 2.82 (0.76)	−47.34 (128)	.001	−4.20	[−5.63, − 5.17]
High benefit	*M*_A4_ = 2.60 (0.90)				
Random victim	*M*_A5_ = − 0.22 (1.80)	10.31 (128)	.001	8.53	[1.57, 2.32]
Specific victim	*M*_A6_ = − 2.18 (1.30)				

*Note*: Paired *t*-tests were controlled for False Discovery Rate (Benjamini–Hochberg) correction for multiple comparisons and remained significant (*p* = .001).

## Reaction scenario

### What arguments do subjects prefer when confronted by an angry individual?

The reaction scenario was constructed as a mirror image of the argument scenario to provide converging evidence to the investigated predictions. The arguments were rewritten, placing the participant in the role of victim on whom the cost was imposed (this cost was a trade-off for a benefit for a friend). The participant had to rate how much each argument would weaken or strengthen their anger. The results confirmed what was expected: the arguments preferred by the reaction scenario participants were those with the potential to reduce the magnitude of their anger if they believed the statement.

As found in the argument scenario, participants preferred arguments stating a high WTR – low cost (Argument 1), high benefit (Argument 4), and random victim (Argument 5) – and rejected those stating a low WTR towards the victim – high cost (Argument 2), low benefit (Argument 3), and specific victim (Argument 6) – as shown in Table 2. The results provide convergent evidence to the predictions tested in this study and in Sell et al. (2017).

**Table 2.** Anger magnitude by reaction scenario

Group	Mean (SD)	*t* (*df*)	*p* adjusted	*d*	CI (95%)
Low cost	*M*_A1_ = – 0.93 (1.80)	− 9.63 (84)	.001	1.05	[−3.03, − 1.99]
High cost	*M*_A2_ = 1.59 (1.90)				
Low benefit	*M*_A3_ = 2.32 (1.27)	21.96 (84)	.001	−2.39	[4.13, 4.95]
High benefit	*M*_A4_ = – 2.22 (1.34)				
Random victim	*M*_A5_ = – 0.57 (1.57)	−11.07 (84)	.001	−1.20	[−2.48, − 1.72]
Specific victim	*M*_A6_ = 1.54 (1.31)				

*Note*: Paired *t*-tests were controlled for False Discovery Rate (Benjamini–Hochberg) correction for multiple comparisons and remained significant (*p* = .001).

Results for the additional six emotions are presented in the supplementary material.

## Discussion of Replication Analysis

The Replication Analysis sought to carry out a direct replication of Sell et al. (2017). We used the same predictions developed in the original study, the same scenarios, and the same experimental manipulations. We expected to fully replicate the original study because we were confident that the information processing mechanisms of anger are distributed in a species-typical manner and, thus, presenting evidence that these mechanisms are psychological adaptations. The six predictions derived from the Recalibrational Theory of Anger tested and confirmed by Sell et al. (2017) in five industrialized cultures and a non-industrialized one, were also confirmed in Brazil. An independent replication in a seventh culture provides additional evidence to the Recalibrational Theory of Anger, demonstrating the universality of anger processing and the functional specialization of this emotion, as predicted for EPM with species-typical distribution (Tooby & Cosmides, 2008, 2015).

While original studies in psychology are commonly not replicable in other laboratories or cultures (Stevens, 2017), our study demonstrates that, even though it was carried out by researchers independent of the original study, the results we found fully replicated those found by Sell et al. (2017), including the effect sizes of large magnitude.

Therefore, with Extension Analysis, we sought to advance our understanding of the emotion of anger by analysing how much its magnitude can be predicted by individual differences related to antisocial personality.

## Results of Extension Analysis

### Initial anger scores and Dark Triad

In the Extension Analysis, our main objective was to examine whether changes in anger scores could be predicted by scores in the Dark Triad domains, i.e., individuals scoring higher on Machiavellianism and/or narcissism, compared with those scoring lower, would exhibit greater change in magnitudes of anger in specific experimental conditions. During the review process, one of the reviewers suggested an additional analysis of the initial anger scores. We agree that this analysis provides new insights into the role of the Dark Triad in anger magnitude, as effects can be assessed both before and after the experimental manipulation.

We found that the regression model for initial anger in the Telephone vignette was significant, with narcissism emerging as a positive predictor; however, the effect size was rather small (adjusted *R*^2^ = .06). The regression model for initial anger in the Lunch vignette was not significant. The results of these analyses are detailed in the SM.

## Change in anger scores and Dark Triad

The single multiple regression with main and interaction effects for the Benefit conditions yielded a significant result (*F* = 3.410; *p* = .002, adjusted *R*^2^ = .07); however, only the main effects of the experimental conditions were significant (*β* = .25, 95% CI [.12, .38], SE = .20; *t* = 3.827, *p* = .001). There were no significant effects for Machiavellianism (*β* = .10, 95% CI [−.10, .30], SE = .30; *t* = 0.964, *p* = .336), Narcissism (*β* = .13, 95% CI [−.06, .33], SE = .43; *t* = 1.375, *p* = .171), or Psychopathy (*β* = − .01, 95% CI [−.22, .19], SE = .36; *t* = − 0.098, *p* = .922). Furthermore, no significant interaction effects were found between the experimental conditions and the scores of Machiavellianism (*β* = .03, 95% CI [−.18, .23], SE = .43; *t* = 0.246, *p* = .806), Narcissism (*β* = − .12, 95% CI [−.31, .07], SE = .60; *t* = − 1.230, *p* = .220), or Psychopathy (*β* = .04, 95% CI [−.17, .24], SE = .51; *t* = 0.359, *p* = .720).

The single multiple regression with main and interaction effects for the Victim conditions also yielded a significant result (*F* = 20.500; *p* = .001, adjusted *R*^2^ = .38), with significant main effects for the experimental conditions (*β* = − .53, 95% CI [−.64, − .43], SE = .17; *t* = − 10.090, *p* = .001) and Machiavellianism (*β* = .21, 95% CI [.03, .38], SE = .26; *t* = 2.826, *p* = .023). No significant effects were found for Narcissism (*β* = − .01, 95% CI [−.17, .14], SE = .35; *t* = − 0.167, *p* = .867) or Psychopathy (*β* = − .05, 95% CI [−.24, .13], SE = .33; *t* = − 0.567, *p* = .571). However, an interaction effect was found between Narcissism scores and the Specific Victim condition (*β* = .20, 95% CI [.04, .36], SE = .49; *t* = 2.490, *p* = .014). No interaction effects were found between the experimental conditions and Machiavellianism (*β* = − .02, 95% CI [−.19, .16], SE = .36; *t* = − 0.216, *p* = .829) or Psychopathy (*β* = .004, 95% CI [−.18, .19], SE = .43; *t* = 0.043, *p* = .966).

## Discussion of Extension Analysis

Results showed that none of our predictions about the dark personality were supported. The experimental conditions emerged as the main predictors of change in anger magnitude across both Benefit and Victim conditions. However, Machiavellianism was a predictor of greater change in anger magnitude in the Lunch vignette, but not when the offender’s benefit is low as we have predicted (Prediction 7). Additionally, an interaction effect was found between Narcissism and Specific Victim condition, contrary to our Prediction 10. The interaction terms were critical to test our predictions of Extension Analysis, but none was supported.

Nevertheless, during the review process one reviewer noted that, given the nature of narcissism, higher narcissism might be associated with greater anger when the victim was specifically targeted and the offender gained only a small benefit – a pattern opposite to our Predictions 8 and 10. This interpretation aligns not only with the Dark Triad literature but also with the Recalibrational Theory, which predicts that individuals who believe they deserve better treatment (e.g., narcissists) will respond with greater anger. After careful consideration, we agreed with this reasoning. Indeed, the interaction between narcissism and the ‘specific victim’ condition is the only result that confirms an effect of Dark Triad traits on anger change scores in our study.

Based on the adaptationist perspective of personality, which interprets intrapersonal, interpersonal, and transcultural variations as results of information processing by EPMs and their impact on fitness (Sznycer & Lukaszewski, 2019), we sought to investigate how individual differences influence the anger magnitude. Thus, Dark Triad was analysed due to its traits’ tendency to maximize personal well-being at the expense of those of others (Lukaszewski et al., 2020). Despite sharing specific characteristics, Dark Triad traits have central characteristics that can influence the expression of emotional reactions (Jones & Paulhus, 2010). Several studies indicate that the antisocial personality domains are associated with self-interested, selfish behaviours, which demonstrate the goal of self-favour, as well as attitudes that reveal negligence or disregard for the well-being of third parties. A core characteristic of the Dark Triad is its negative WTR for social partners (Furnham et al., 2013; Koehn et al., 2019; Lukaszewski et al., 2020). Individuals with greater traits of Dark Triad were expected to display greater anger magnitude in specific experimental conditions. However, we found this only in the interaction between Narcissism and Specific Victim condition.

## General discussion

The results of Replication Analysis and Extension Analysis are quite contrasting. While we were able to successfully replicate all the predictions derived from the Recalibrational Theory of Anger, predictions which Sell et al. (2017) previously tested, only one result suggested an effect of Dark Triad, i.e., Narcissism, on anger change scores. Regarding Replication Analysis, it is important to highlight that the results were replicated even with scenarios that described situations that are not necessarily usual and recurring for the participants, such as being in line at a public telephone, since the use of public telephones fell into disuse with the advancement of smartphones. It is noteworthy that this occurred even when we used scenarios that could be distant from the contexts of the participants, confirming the cognitive strength, as well as the flexible and regulatory nature of the EPM information processing (Tooby & Cosmides, 2015).

The findings in Part 1 of the Telephone and Lunch vignettes confirm the functional design of the emotion of anger (Sznycer et al., 2021) since, even before the manipulation of costs and benefits, the information processing of this emotion was triggered in front of the devaluation cues described in the scenarios – in addition, this also demonstrates the functionality of the experimental vignettes. Part 2 of these vignettes tested, through the manipulation of benefits and specificity of victims, the triggers of anger, i.e., how information about the magnitude of the benefit obtained, and the identity of the victim influenced the processing of anger information. Prediction 1, which states that ‘holding constant the benefit received by the offender, anger will become more intense as the cost imposed on the offended individual increases’, was not tested by either Sell et al. (2017) or by our study, since it had been demonstrated previously. However, we found that the anger magnitude increases when information indicates offender’s benefit was small rather than large (Prediction 2), and that anger is greater when the offender intentionally, rather than randomly, imposed the cost (Prediction 3), as proposed by Sell et al. (2017).

Argument/reaction vignettes confirmed Predictions 4–6, testing which arguments individuals prefer when confronting an offender or confronted by an angry individual, according to the point of view that people make implicit calculations of well-being compensation (Delton & Robertson, 2016). Previous findings support the hypothesis that people use information about an offender’s intentions (Berkowitz & Harmon-Jones, 2004) to evaluate the offender’s WTR over the victim by evaluating the relationship between costs and benefits. When someone imposes a cost on another, the trade-off of costs and benefits indicates the offender’s WTR over the victim. When the imposition of a cost reveals a low WTR of one individual over another, the processing of information about the emotion of anger is activated in an attempt to recalibrate this relationship (Sell et al., 2017).

The qualitative data obtained provides additional evidence that strongly supports the investigated predictions. As in the original study, participants who responded to the low benefit condition reported that their anger increased when they saw a cost in exchange for a small benefit: ‘It increased, because, for me, it’s not worth pushing someone else and making them miss the bus for just a 5 reais ticket.’ In contrast, in the High Benefit condition, the anger subsided upon learning that the cost resulted in a great benefit: ‘It decreased because compared to the consequence of me not using the phone and missing the bus, it is less harmful than his, which would be losing 5thousand.’ In the specific victim context, anger increased upon learning that the offense was intentional: ‘Yes, because it wasn’t just a joke, it was also targeted [i.e., purposefully directed at the victim].’ Meanwhile, in the random victim condition, the anger subsided upon discovering that the offense was random: ‘It decreased, therefore, for I would now be aware that the joke was not directed at me specifically’ (underline indicates our emphasis on selected reports from participants). Consequently, the participants’ reports also indicate that (i) the size of the benefit obtained by the offender and (ii) the intentionality of the offensive act are both aggravating/mitigating factors for the offense’s seriousness, signalling greater or lesser devaluation and triggering greater or lesser anger. The SM provides these results in detail.

Concerning the working hypothesis of the Extension Analysis, we predicted that there would be individual differences in the magnitude of anger promoted by the Dark Triad domains. As mentioned, individuals with higher levels of these traits present self-interested behaviours and attitudes that reveal an inclination to favour themselves over others in situations of conflict of interest. From studies of emotions, the search for personal favour can be interpreted as a WTR with other individuals. Individuals high in Machiavellian traits were predicted to feel more anger in situations where they suffer a cost for a low benefit (Prediction 7) or when they are specifically victimized (Prediction 9). These contexts signal a low WTR of the offender, which goes against the Machiavellian strategic orientation, whose formation of alliances requires a perception of reciprocity and cooperation, characteristics of high WTRs (Delton & Robertson, 2016).

In parallel, we predicted that individuals with higher Narcissism would present greater change magnitude of anger in the large benefit condition (Prediction 8) and in the random victim condition (Prediction 10). These situations indicate a high WTR of the offender over the victim and a potential for more cooperative relationships (Delton & Robertson, 2016). It is expected that narcissists would prioritize their well-being so strongly that any imposition of cost would be a great personal offense, given their high sensitivity to self-image (Koehn et al., 2019). Precisely for this reason, it makes more sense – both according to the Dark Triad theory and the Recalibrational Theory – that narcissists would feel angrier under conditions opposite to our predictions (low benefit and specific victim). We acknowledge that our Predictions 8 and 10 were not perfectly aligned with the theories tested. However, our results did not reveal narcissism as a significant predictor in the Low Benefit condition. Differently, Narcissism only predicted change in anger magnitude in the Specific Victim condition, perhaps because the sense of entitlement of individuals with higher levels of narcissism was threatened by others who intended to target them directly.

Therefore, the set of results suggests that anger processing is robust and has limited influence from the Dark Triad, which can be explained by the invariant and universal design of EPMs. It is reasoned that the scenarios described social situations of conflicts of interest that represent such strong environmental challenges that they robustly activated the cognitive processing of anger so that individual differences were unable to exert a great influence in the investigated context. With this study, we cannot rule out that the magnitude of anger varies depending on other individual differences, such as other personality traits, self-esteem, etc. On the contrary, emotional processing of emotions predicts that internal regulatory variables (e.g., self-esteem, partner value, relatedness index) will influence emotional processing (Delton & Robertson, 2016; Tooby & Cosmides, 2015). However, precisely for that reason, the absence of a relationship between Dark Triad and the magnitude of anger, which is theoretically predicted, is a surprising finding that deserves further testing.

It is important to highlight that the instrument used to measure Dark Triad presented psychometric limitations that may have influenced the results. Although SD3 is recommended for joint assessment of Dark Triad Personality domains and has been adapted for several countries (Maples et al., 2014), its psychometric properties still require in-depth exploration. In the Brazilian context, Monteiro (2017) adapted SD3, identifying limitations regarding factorial, convergent validity, and internal consistency. Despite these limitations, SD3 stands out, especially for being a concise measure that minimizes participant fatigue but manages to address central elements of each construct, in addition to presenting evidence of consistent incremental and convergent validity (Monteiro, 2017). However, the factorial structure of SD3 requires adjustments in different contexts, especially in the number of items (Maples et al., 2014; Monteiro, 2017), as we also observed in this study.

In our models, despite acceptable fit indices, modification indices suggested the removal of several items from the domains of Machiavellianism (five items retained) and narcissism (six items retained), while the Psychopathy domain lost only one item (eight items retained). Furthermore, several items presented cross-domain explanations, in addition to those theoretically expected, as is the case with the Narcissism scale item. The high correlation between Machiavellianism and Psychopathy also suggests significant shared variance between these domains.

Considering the limitations observed in the Brazilian version of SD3, the instrument did not fully capture the construct of antisocial personality. Although Monteiro (2017) suggests the use of SD3 with reservations, in the absence of more appropriate psychometric tools, we believe it is essential to test the functionality of SD3 in our study. Therefore, future studies must develop more psychometrically appropriate instruments to confirm our findings.

The results of this research deepen the understanding of social emotions and personality, highlighting the universality of EPMs and their interaction with the environment and individual differences. Direct replication, carried out with rigorous experimental control in a heterogeneous, cross-cultural sample, strengthens the external validity of the functional model of anger. An evolutionary perspective highlights the adaptive character of dark traits, explaining their persistence and interaction with contemporary social factors. Given the antisocial potential of these traits, understanding the underlying mechanisms of Dark Triad and its relationship to anger may aid in the development of interventions to mitigate antisocial behaviours.

Future research should use more advanced statistical methods and consider other individual variables. These findings expand the external validity of the Recalibrational Theory of Anger and offer insights into the dynamics of social conflicts and mediation of aggressive behaviours, in addition to stimulating scientific replication in the area.

## Supporting information

10.1017/ehs.2025.10030.sm001 Righi and Silva Júnior supplementary material Righi and Silva Júnior supplementary material

## Supplementary material

The supplementary material for this article can be found at https://doi.org/10.1017/ehs.2025.10030.

## Research transparency and reproducibility

The data that support the findings of this study are available in the Open Science.

Framework (OSF) repository: https://osf.io/z9yra/?view_only=13906f2284bb4e27a535d566aa486978
