# Supplementary material for: Comparing predictions of anger in conflict situations: Recalibrational Theory versus Dark Triad traits
Source: Evol Hum Sci. 2025 Dec 23;8:e22. doi: 10.1017/ehs.2025.10030 (PMC13227133; doi:10.1017/ehs.2025.10030)
Supplement: Righi and Silva Júnior supplementary material [file S2513843X25100303sup001.docx]

**Supplementary Material**

**Experimental Controls**

**Pilot**

In order to evaluate the experimental controls, response time, and seek refinements and improvements in the instrument, a pilot study was conducted prior to data collection. The pilot was carried out on the SurveyMonkey platform using the same instruments present in the final form. A total of 20 responses were collected online and remotely, from a convenience sample.

Control configurations were set up in the form, created on the SurveyMonkey platform, and tested and validated in the pilot study. All questions, except those in the thank-you section, were marked as mandatory to prevent incomplete responses. After reading the Informed Consent Form, participants could click "I agree to participate" to express their consent.

It was configured and verified that the Short Dark Triad (SD3) and the experimental vignettes were presented in a counterbalanced order to control for order effects. For the random assignment of participants to the experimental conditions of Telephone and Lunch vignettes, the "A/B Test" feature of the SurveyMonkey platform was used, ensuring that 50% of the sample responded to each experimental condition. This configuration was feasible because each condition consisted of two texts followed by a common set of questions. In the Telephone vignette, 50% responded to the High Benefit Condition (HBC) and 50% to the Low Benefit Condition (LBC); in Lunch vignette, the distribution was between Random Victim and Specific Victim.

In Argument/Reactions vignette, composed of two scenarios —Argument Scenario or Reaction Scenario— with distinct texts and responses, a forced-choice question was implemented to randomly assign participants. Participants were instructed to choose between "qwty" or "ytwq", sequences created by the researchers to generate random allocation for argument scenario and reaction scenario, respectively. The pilot validation with the convenience sample indicated that participants chose randomly, without any influence on their choice.

Attention questions were tested and considered clear, with grammatical adjustments made. The average response time was set at 15 minutes, as observed in the pilot study.

**Attention Questions**

In Telephone and Lunch vignettes, participants were instructed to read the described scenarios and imagine the magnitude of anger and other emotions they might feel if they experienced the described story. Initially, they were to read the story from part 1 and indicate the magnitude of the imagined anger. Then, as they proceeded to the next page of the form, a control setting was implemented to prevent them from returning to the previous page and re-reading the story. To verify whether participants read part 1 attentively, part 2 of the scenarios included an attention question with response options. Both questions addressed a crucial aspect of part 1 related to the cost imposed on the participant, which is essential for understanding the subsequent experimental conditions: in Telephone vignette, it referred to the waiting time for the bus, and in Lunch vignette, to the phrase marked on the lunch packaging.

**Replication Analysis**

**Results**

**Influence of Response Modality of Participants**

Conducting the research in both in-person and online modalities allowed for an assessment of whether the environment in which participants answered the survey influenced their performance. No significant difference was found in Telephone vignette, MEFR_online_ = 5.42 (*SD* = 2.30); MEFR_in-person_ = 5.50 (*SD* = 2.20); *t*(223) = -0.26, p = .80, 95% CI [-0.76, 0.58], nor in Lunch vignette, MEFR_online_ = 7.02 (*SD* = 2.14); MEFR_in-person_ = 7.24 (*SD* = 1.90); *t*(223) = -0.71, *p* = .47, 95% CI [-0.83, 0.39].

**Attention Questions**

It was also analyzed whether the mean scores of participants in FAS differed significantly between all participants and only those who answered the attention questions correctly in Telephone vignette and Lunch vignette. In Telephone vignette, 16 people answered incorrectly, while in Lunch vignette, 41 failed the attention question. No significant differences were found between the groups, allowing for a joint analysis. In Telephone vignette, *t*(223) = 0.81, *p* = .41, 95% CI [-0.67, 1.63]; in Lunch vignette, *t*(223) = 1.88, *p* = .06, 95% CI [-0.03, 1.37].

**Do Dark Triad Traits predict Initial Anger Scores?**

During the review process, one of the reviewers suggested examining whether the scores of the Dark Triad domains predicted the *initial* anger scores. Although these analyses were not part of the original plan, they offer potentially valuable insights for our study. The results are presented below.

**Telephone vignette**

The multiple regression for the initial anger score for the Telephone vignette yield a significant result (*F* = 5.848; *p* = .001, adjusted *R²* = .06) demonstrating that Narcissism showed a significant effect (*β* = .145, SE = .273; *t* = 2.151, *p* = .033), whereas Machiavellianism did not (*β* = .145, SE = .199; *t* = 1.880*, p* = .061) or Psycopathy (β = .075, SE = .235; *t* = 1.003*, p* = .317).

**Lunch vignette**

The multiple regression for the initial anger score for the Lunch vignette was non-significant (*F* = 1.826; *p* = .143, *R²* adjusted = .011).

**Other Emotions**

**Telephone vignette**

**Does the information that the benefit the offender receives, in the face of imposing a cost, is high or low reflect other emotional responses beyond anger?**

As in Sell et al.’ (2017) study, participants were asked whether they perceived other emotions, besides anger, when reading the scenarios described in Telephone and Lunch vignettes, to rule out alternative explanations. Independent t-tests were conducted between the experimental conditions (High Benefit Condition and Low Benefit Condition) to determine if there were significant differences in the means of the participants concerning the emotions of happiness, surprise, sadness, fear, envy, shame, and compassion. As in Sell et al.’ (2017) study, results indicated significant differences for “happiness,” “surprise,” “envy,” and “compassion”. Table SM1 reflects the data found in Telephone vignette.

These findings suggest explanations associated with the size of the benefit received by the offender in relation to the imposition of a cost. Participants reported higher levels of happiness, surprise, envy, and compassion in High Benefit Condition compared to Low Benefit Condition. This may be interpreted as an indication that when a victim faces a cost in exchange for a significant benefit to the offender, the magnitude of that benefit may mitigate the anger emotion, reducing the perception of devaluation.

The Recalibrational Theory of Anger (RTA) proposes that this emotion can be alleviated when the "self" recognizes that the "other" can provide valuable resources, which is beneficial for fitness (Von Rueder et al., 2008). The perceived magnitude of anger in High Benefit Condition was M_anger_ = -.86 (SD = 1.53), in this condition, the offenders receive a large sum of money, and the victim suffers the cost of waiting another hour for the bus. It is inferred that implicit emotional processing may also be related to the value of the financial resource received, not just the signaling of devaluation.

**Table SM1**

*Statistical Values of Other Emotions in Telephone vignette*

*Note.* Emotions in bold indicate statistically significant differences between group means in High Benefit

| **Emotion** | **Group** | **N** | **M (SD)** | ***t* (df)** | ***p*** | **95% (CI)** |
| --- | --- | --- | --- | --- | --- | --- |
| **Happiness** | High Benefit | 110 | 2.32 (1.63) | 3.92 (223) | .001 | [0.37, 1.14] |
|  | Low Benefit | 115 | 1.56 (1.26) |  |  |  |
| **Surprise** | High Benefit | 110 | 4.36 (2.06) | 2.80 (223) | .006 | [0.22, 1.31] |
|  | Low Benefit | 115 | 3.58 (2.07) |  |  |  |
| Sadness | High Benefit | 110 | 2.43 (1.79) | 0.73 (223) | .468 | [-0.67, 0.31] |
|  | Low Benefit | 115 | 2.61 (1.94) |  |  |  |
| Fear | High Benefit | 110 | 1.45 (1.23) | 0.01 (223) | .989 | [-0.28, 0.29] |
|  | Low Benefit | 115 | 1.44 (0.98) |  |  |  |
| **Envy** | High Benefit | 110 | 2.91 (1.86) | 6.26 (223) | .001 | [0.92, 1.76] |
|  | Low Benefit | 115 | 1.57 (1.33) |  |  |  |
| Shame | High Benefit | 110 | 1.86 (1.67) | 0.45 (223) | .652 | [-0.30, 0.48] |
|  | Low Benefit | 115 | 1.77 (1.27) |  |  |  |
| **Compassion** | High Benefit | 110 | 3.14 (2.06) | 3.92 (223) | .001 | [0.47, 1.43] |
|  | Low Benefit | 115 | 2.18 (1.57) |  |  |  |

Condition and Low Benefit Condition.

As in Sell et al.’ (2017) study, we run a multiple regression model using the experimental manipulation and the emotions of happiness, surprise, envy, and compassion on the change in anger. Like the original study, the experimental manipulation remained a significant predictor of the subject’s change in anger even controlling for the other emotions (*F* = 20.724; *p* = .001, *R²* adjusted = .31, β = -.145, SE = .194; *t* = -2.302*, p* = .022). Alongside the experimental manipulation, the emotions of happiness (β = -.36, *p* = .001) and compassion (β = -.27, *p* = .001) were significant predictors. However, the emotions of envy (β = .07, *p* = .259) and surprise (β = .06, *p* = .327) were not significant predictors. As in the Sell et al.’ study, participants who felt happier or more compassion about the target’s lottery winnings experienced a decrease in their anger.

At first glance, these results may appear to contradict our predictions, given that the magnitude of the change in anger scores can be predicted by the emotions of happiness and compassion. However, it is important to note that in complex social situations, such as this one, different emotions can be co-activated (Sznycer et al., 2016, 2017). Thus, feeling pleased about the offender’s success may be associated with lower levels of anger, but not with its elimination when a cost has been imposed. Prediction 2 anticipates precisely this outcome (holding the imposed cost constant, anger will become less intense as the benefit received by the offender increases). Therefore, the association of happiness and compassion with the reduction in the magnitude of anger in the high-benefit condition is consistent with the Recalibration Theory.

**Lunch vignette**

**Does the information that the cost imposed was random or deliberate on the victim trigger other emotions beyond anger?**

Independent t-tests were conducted between the experimental conditions (random victim and specific victim) to determine whether there were significant differences in the participants’ mean ratings for the emotions of happiness, surprise, sadness, fear, envy, shame, and compassion. The emotions "happiness," "surprise," and "sadness" differed significantly between the random victim and specific victim conditions. Table SM2 reflects the data found in Lunch vignette.

**Table SM2**

*Statistical Values of Other Emotions in Lunch vignette*

| **Emotion** | **Group** | **N** | **M (SD)** | ***t*(df)** | ***p*** | **CI (95%)** |
| --- | --- | --- | --- | --- | --- | --- |
| **Happiness** | Specific Victim | 117 | 1.09 (0.60) | -2.41 (223) | .002 | [-0.53, -0.05] |
|  | Random Victim | 108 | 1.38 (1.17) |  |  |  |
| **Surprise** | Specific Victim | 117 | 4.30 (2.22) | 3.40 (223) | .001 | [0.42, 1.60] |
|  | Random Victim | 108 | 3.29 (2.25) |  |  |  |
| **Sadness** | Specific Victim | 117 | 4.96 (1.94) | 2.68 (223) | .008 | [0.19, 1.24] |
|  | Random Victim | 108 | 4.24 (2.08) |  |  |  |
| Fear | Specific Victim | 117 | 3.03 (2.15) | 1.72 (223) | .088 | [-0.07, 1.05] |
|  | Random Victim | 108 | 2.55 (2.12) |  |  |  |
| Envy | Specific Victim | 117 | 1.31 (1.00) | 1.04 (223) | .301 | [-0.11, 0.36] |
|  | Random Victim | 108 | 1.19 (0.75) |  |  |  |
| Shame | Specific Victim | 117 | 4.35 (2.32) | 1.01 (223) | .314 | [-0.31, 0.95] |
|  | Random Victim | 108 | 4.03 (2.48) |  |  |  |
| Compassion | Specific Victim | 117 | 1.52 (1.42) | -1.53 (223) | .129 | [-0.72, 0.09] |
|  | Random Victim | 108 | 1.83 (1.65) |  |  |  |

*Note*. Emotions in bold indicate statistically significant differences between group means in Specific Victim Condition and Random Victim Condition.

Participants reported higher levels of "surprise" and "sadness" in the specific victim condition, while in the random victim condition, they perceived greater "happiness." These results suggest explanations related to the effect of the intentionality of the target in relation to the imposition of a cost. It is worth noting that in the specific victim condition, the average magnitude of anger was significantly higher M_anger_= 8.02, SD = 2.04) than the means of the emotions of surprise and sadness, indicating a robust processing of anger in response to the imposition of a cost associated with the information of being a specifically targeted victim, a strong cue of devaluation. However, the emotions of sadness and surprise may also be related to the intentionality information about the victim. In the random victim condition, the magnitude of anger was also considerably higher (M_anger_ = 6.07, SD = 1.58), but the processing of the emotion of happiness may have been triggered by the information about the randomness of the victim, suggesting a sense of "relief" for not having suffered an intentional cost.

**Qualitative Data**

**Telephone vignette**

**How can we determine if participants made an implicit calculation of the Welfare Tradeoff Ratio (WTR) of the offender towards the victim?**

In the in-person data collection, the question "Did the anger you would feel in this moment change (increased or decreased) compared to your previous response? If so, please explain in your own words why" was added to part 2 of the experimental scenarios Telephone vignette and Lunch vignette to investigate the processing of the anger emotion information.

The results support the theory that individuals make implicit WTR calculations in decision-making processes (Delton & Robertson, 2016). The emphasis indicates that the size of the benefit obtained by the offender serves as an aggravating factor of the offense, as it provides a cue for greater or lesser devaluation. When the benefit is low, anger increases; on the other hand, when it is high, anger tends to decrease.

In the Low Benefit condition most individuals reported feeling more anger or the same level of anger upon discovering they had suffered a cost in exchange for a small benefit (Table SM3). Only a minority of participants indicated that their anger decreased after learning the reason behind the cost they had incurred.

**Table SM3**

*Distribution of Participants by Experimental Condition and Direction of Self-Reported Anger Change*

| **Condition** | **N** | **Anger Increased** | **Anger Unchanged** | **Anger Decreased** |
| --- | --- | --- | --- | --- |
| Low Benefit | 31 | 13 | 12 | 6 |
| High Benefit | 30 | 9 | 0 | 19 |
| Specific Victim | 34 | 25 | 9 | 0 |
| Random Victim | 27 | 6 | 10 | 11 |

Some of the arguments for the increased anger were: "It increased because, for me, it's not worth pushing someone else and making them miss the bus for just a 5 *reais* ticket"; "It increased because it wasn't even a high amount"; "It increased a little because I don't know what his needs were to want so desperately a 5 *reais* ticket to the point of pushing someone"; "On one hand, I understand, he's excited to have won, but it's still 5 *reais*. He worsened my day exponentially because of something that only improved his day a little"; "The anger increased considering the man's motivation to push me out of the phone line to make his own call. In my view, being rude and disrespectful to someone who was waiting respectfully in line is an attitude of disrespect, especially when the intentions behind the action reveal themselves to be motivated by greed for money, and especially for such a small amount like R$5." (emphasis added).

In the high benefit condition, most individuals reported feeling less anger or the same level of anger when they learned that they had incurred a cost in exchange for a large benefit for the other person (Table SM3). Only a small number of participants reported that their anger decreased after learning the reason why they had incurred the cost.

Some of the arguments for the decrease in anger were: "It decreased because his situation was more urgent than mine"; "It decreased because, compared to the consequence of me not using the phone and missing the bus, it was less damaging than his, which would be losing 5 thousand"; "Yes, because the man had a plausible reason to interrupt me"; "The anger decreased because I believe he really needed the money and it was important for him"; "It decreased because I realized he also had an urgency like mine, and if he lost that opportunity, he wouldn't get another chance, unlike me, who can wait for the bus"; "It decreased because, although he pushed me, he had a reason for it. Not that his need was above mine, but at least I know he didn't do it for nothing, although I would call his attention if he tried to walk away as if nothing had happened without apologizing."

**Lunch vignette**

**How can we determine if participants made an implicit calculation of the Welfare Tradeoff Ratio (WTR) of the offender towards the victim?**

As in Telephone vignette, during the in-person data collection, the question "Did the anger you would feel in this moment change (increased or decreased) compared to your previous response? If so, please explain in your own words why" was added to part 2 of Lunch vignette to investigate the processing of anger emotion information.

The results in this scenario also support the theory that individuals make implicit WTR calculations in decision-making processes (Delton & Robertson, 2016). The emphasis indicates that the offender's intention acts as an aggravating factor for the offense. When the intention is perceived, anger increases; on the other hand, in its absence, anger tends to decrease.

In the specific victim condition, most individuals reported feeling more anger or the same level of anger when they learned that they had incurred a cost in exchange for a large benefit for the other person (Table SM3). No participants reported a decrease in anger. Some justifications for the increased anger included: "He did it on purpose"; "It increased because now I believe it wasn't just because he’s a bad person, I think it’s personal"; "It increased because it's clear that it wasn't just a joke, it was something intentional, maybe because he doesn't like me or for revenge"; "It increased because it was something personal to harm me"; "Yes, because he did something to specifically affect me negatively"; "Yes, because it wasn't just a joke, it was also directed at me"; "He knew it was my lunch, he could have used anyone's, but it seemed personal"; "It was a calculated humiliation, not just a random attack, but directed, which makes the situation more personal and worse"; "Yes, my anger increased simply because João knew from the beginning that the lunch was mine" (emphasis added).

In the random victim condition, most individuals reported feeling less anger or the same level of anger when they learned that they had suffered a cost in exchange for a large benefit for the other person (Table SM3). Six participants reported increased anger. Some justifications for anger decrease or remained the same included: "João simply chose a random person to make the 'joke' (someone he doesn't have an intimate relationship with)"; "It decreased because I now know that the joke wasn’t directed at me specifically"; "It changed a bit, because this would make me take it less personally, feel less embarrassed, and realize that the problem wasn't with me, but with the person who made such a tasteless joke. Despite that, I would still feel a lot of anger because, regardless of whether it was me or someone else in this situation, this kind of behavior is absurd and cruel"; "It decreased because it wasn’t a directed attack at me, but I would still be angry that someone would put something disgusting in my food just to amuse others"; "Because I would see that it wasn't personal, just a joke"; "Yes. The anger decreased just a little, considering that João's intention wasn't to harm or joke about me specifically, but it still consists of an unnecessary, unethical, and disrespectful attitude towards anyone"; "Because I would see that it wasn’t personal, just a joke"; "It decreased a little, because it didn’t seem personal"; "It would decrease a little because now I know it wasn't a provocation directed at me" (emphasis added).

**Extension Analysis**

**Data Analysis Procedures**

A series of confirmatory factor analyses were conducted to assess the plausibility of a multidimensional structure for the Short Dark Triad (SD3; Jones & Paulhus, 2014). The analysis was implemented using the Weighted Least Square Mean and Variance Adjusted (WLSMV) estimation method, suitable for categorical, ordinal, or non-normal data (DiStefano & Morgan, 2014; Li, 2016).

The fit indices used were: χ²; χ²/df; Comparative Fit Index (CFI); Tucker-Lewis Index (TLI); Standardized Root Mean Residual (SRMR); and Root Mean Square Error of Approximation (RMSEA). χ² values should not be significant; the χ²/df ratio should be < 5 or, preferably, < 3; CFI and TLI values should be > .90 and, preferably, above .95; RMSEA values should be < .08 or, preferably, < .06, with a confidence interval (upper limit) < .10 (Brown, 2015).

**Results**

The multidimensional structure showed satisfactory fit results. As shown in Table SM4, the chi-square values were significant, while the chi-square to degrees of freedom ratio was also below the recommended threshold (1.797). The CFI, TLI, RMSEA, and SRMR indices supported the model. To further inspect the results, modification indices were evaluated, revealing high residual covariance between some item pairs of the Narcissism factor and between items explained by factors beyond those theoretically expected. This occurred particularly with the factors of Machiavellianism and Psychopathy explaining items of Narcissism. By adding this residual covariance to the model and removing items with factor loadings < .3, there was an improvement in all fit indices (see Table SM4). The structure and factor loadings of the final model are presented in Figure SM1, while the psychometric properties of the SD3 final model are shown in Table SM5. Table SM6 presents the Short Dark Triad item content and standardized factor loadings.

**Table SM4**

*Fit Statistics for All Estimated Confirmatory Models*

| Model | *χ*² (df) | *χ*²/df | RMSEA [95% CI] | CFI | TLI | SRMR |
| --- | --- | --- | --- | --- | --- | --- |
| Model 1 | 564.919** (321) | 1.760 | .058 [.050, .066] | .82 | .80 | .086 |
| Model 2 | 508.998** (316) | 1.610 | .052 [.044, .060] | .86 | .84 | .080 |
| Model 3 | 486.658** (316) | 1.540 | .050 [.041, .058] | .87 | .86 | .077 |
| Model 4 | 474.151** (312) | 1.520 | .048 [.039, .057] | .88 | .87 | .076 |
| **Model 5** | **258.826**** (**144**) | **1.797** | **.060 [.048, .071]** | **.91** | **.90** | **.074** |

*Note*. *χ*^2^ = chi-squared; df = degrees of freedom; CFI = Comparative Fit Index; TLI = Tucker-Lewis Index; SRMR = Standardized Root Mean Square Residual; RMSEA = Root Mean Square Error of Approximation; ** *p* < 0,001.

Figure SM1 presents the structure and the factor loadings of the items.

**Figure SM1**

*Structure and factor loadings for SD3 final model*


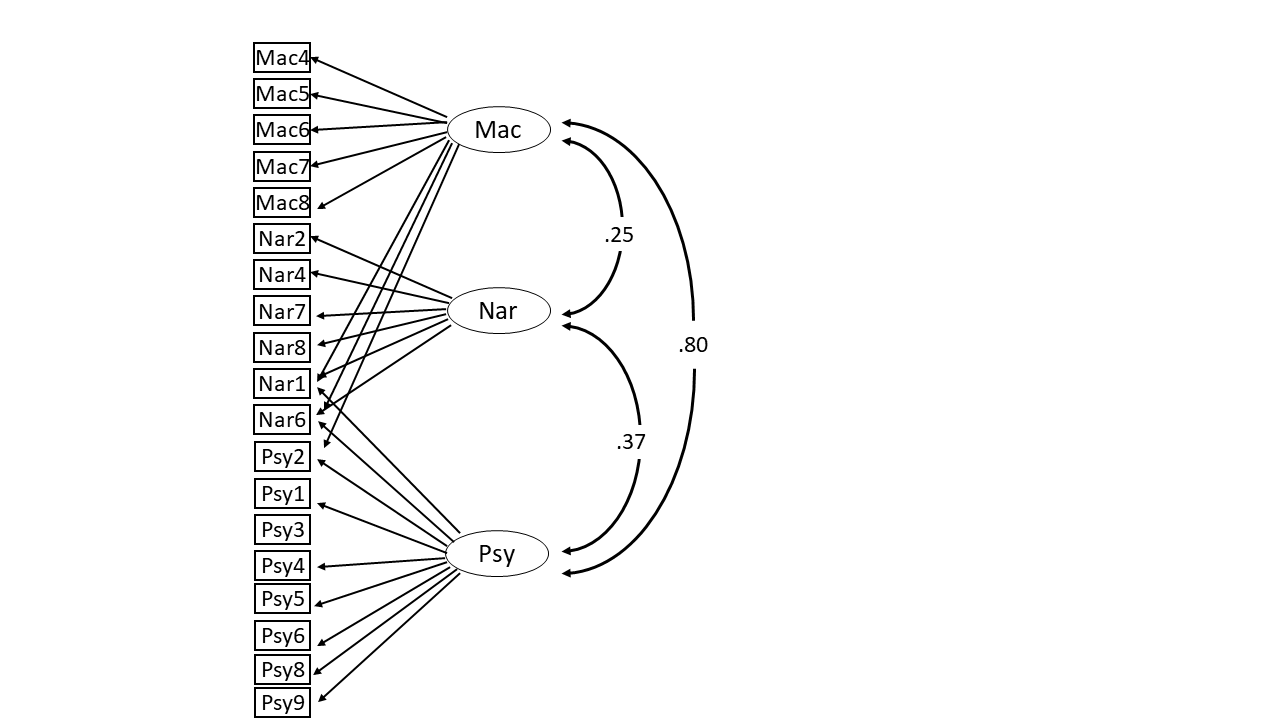


*Note.* Mac = Machiavellianism; Nar = Narcissism; Psy = Psychopathy

**Table SM5**

*Psychometric Properties for SD3 final model*

|  | *M* | *SD* | Range | Composite reliability |
| --- | --- | --- | --- | --- |
| MAC | 2.88 | 0.68 | 1.0-4.6 | .80 |
| NAR | 3.07 | 0.38 | 2.0-4.5 | .63 |
| PSY | 2.16 | 0.51 | 1.3-3.8 | .87 |

*Note.* MAC = Machiavellianism; NAR = Narcissism; PSY = Psychopathy

**Table SM6**

*Short Dark Triad Item Content and Standardized Factor Loadings*

| Item |  | MAC | NAR | PSY |
| --- | --- | --- | --- | --- |
| MAQ4. Avoid direct conflict with others, as they may be useful in the future. |  | .37 |  |  |
| MAQ5. It is wise to keep information that you could later use against people. |  | .76 |  |  |
| **MAQ6 You should wait for the right moment to take revenge on people.** |  | **.87** |  |  |
| MAQ7. There are things you should hide from others because they do not need to know. |  | .35 |  |  |
| MAQ8 Make sure your plans benefit you, not others. |  | .33 |  |  |
| **NAR1 People see me as a natural leader.** |  |  | **.66** |  |
| NAR2 I hate being the center of attention. n |  |  | .50 |  |
| NAR4 I know I’m special because everyone keeps telling me so. |  |  | .46 |  |
| NAR6 I feel embarrassed if someone compliments me. |  |  | .32 |  |
| NAR7 I have been compared to famous people. |  |  | .32 |  |
| NAR8 I am an ordinary person. |  |  | .53 |  |
| PSY1 I like to take revenge on authorities. |  |  |  | .67 |
| **PSY2 I avoid dangerous situations.** |  |  |  | **1.10** |
| PSY3 Getting even needs to be quick and vicious. |  |  |  | .64 |
| PSY4 People usually say that I am out of control. |  |  |  | .45 |
| PSY5 It is true that I can be cruel. |  |  |  | .60 |
| PSY6 People who mess with me always regret it. |  |  |  | .73 |
| PSY8 I enjoy having sex with people I hardly know. |  |  |  | .54 |
| PSY9 I will say anything to get what I want. |  |  |  | .63 |

*Note.* MAC = Machiavellianism; NAR = Narcissism; PSY = Psychopathy. Bolded items represent those with the highest factor loadings in the respective analysis.

**Short Dark Triad**

**INSTRUCTIONS**: Please indicate your degree of agreement or disagreement with the following statements.

1. **Strongly Disagree**
2. **Disagree**
3. **Neither Agree nor Disagree**
4. **Agree**
5. **Strongly Agree**
6. It is not wise to share your secrets.

(Strongly Disagree) 1 2 3 4 5 (Strongly Agree)

1. In general, people do not work hard unless they have to.

(Strongly Disagree) 1 2 3 4 5 (Strongly Agree)

1. At all costs, you must have important people by your side.

(Strongly Disagree) 1 2 3 4 5 (Strongly Agree)

1. Avoid direct conflict with others, as they may be useful in the future.

(Strongly Disagree) 1 2 3 4 5 (Strongly Agree)

1. It is wise to keep information that you could later use against people.

(Strongly Disagree) 1 2 3 4 5 (Strongly Agree)

1. You should wait for the right moment to take revenge on people.

(Strongly Disagree) 1 2 3 4 5 (Strongly Agree)

1. There are things you should hide from others because they do not need to know.

(Strongly Disagree) 1 2 3 4 5 (Strongly Agree)

1. Make sure your plans benefit you, not others.

(Strongly Disagree) 1 2 3 4 5 (Strongly Agree)

1. Most people can be manipulated.

(Strongly Disagree) 1 2 3 4 5 (Strongly Agree)

1. People see me as a natural leader.

(Strongly Disagree) 1 2 3 4 5 (Strongly Agree)

1. I hate being the center of attention.

(Strongly Disagree) 1 2 3 4 5 (Strongly Agree)

1. Many group activities tend to be boring without me.

(Strongly Disagree) 1 2 3 4 5 (Strongly Agree)

1. I know I’m special because everyone keeps telling me so.

(Strongly Disagree) 1 2 3 4 5 (Strongly Agree)

1. I like to meet important people.

(Strongly Disagree) 1 2 3 4 5 (Strongly Agree)

1. I feel embarrassed if someone compliments me.

(Strongly Disagree) 1 2 3 4 5 (Strongly Agree)

1. I have been compared to famous people.

(Strongly Disagree) 1 2 3 4 5 (Strongly Agree)

1. I am an ordinary person.

(Strongly Disagree) 1 2 3 4 5 (Strongly Agree)

1. I insist on getting the respect I deserve.

(Strongly Disagree) 1 2 3 4 5 (Strongly Agree)

1. I like to take revenge on authorities.

(Strongly Disagree) 1 2 3 4 5 (Strongly Agree)

1. I avoid dangerous situations.

(Strongly Disagree) 1 2 3 4 5 (Strongly Agree)

1. Getting even needs to be quick and vicious.

(Strongly Disagree) 1 2 3 4 5 (Strongly Agree)

1. People usually say that I am out of control.

(Strongly Disagree) 1 2 3 4 5 (Strongly Agree)

1. It is true that I can be cruel.

(Strongly Disagree) 1 2 3 4 5 (Strongly Agree)

1. People who mess with me always regret it.

(Strongly Disagree) 1 2 3 4 5 (Strongly Agree)

1. I have never had problems with the law.

(Strongly Disagree) 1 2 3 4 5 (Strongly Agree)

1. I like to annoy losers.

(Strongly Disagree) 1 2 3 4 5 (Strongly Agree)

27. I will say anything to get what I want.

(Strongly Disagree) 1 2 3 4 5 (Strongly Agree)

**Experimental Vignettes**

**Telephone vignette**

**Part 1.**

Read the following description carefully as if you were really in this situation:

Imagine...

You are at the shopping mall. Once you finish, you realize you forgot to call your friend. You promised to call him once you were done. The bus that goes from the mall to the place you agreed to meet departs at exactly 5:00 PM, and it's already 4:50 PM, but you need to call your friend before getting on the bus.

There is a short line for the payphone by the bus stop, and unfortunately, you're not sure if you'll be able to call him before the bus leaves. If you miss the bus, you'll have to wait an hour for the next one, and you don't want to wait all that time. You get in line at the payphone and wait.

Finally, at 4:59 PM, it's your turn to use the phone. If you hurry, you know you can still catch the bus. Just as you’re about to grab the phone, a well-dressed man you know from university pushes you out of line while saying, “Watch out.” He grabs the receiver and dials a number. A second later, you see the bus depart, and you know you'll have to wait for an hour.

**On a scale of 1 to 7, where 1 is not at all and 7 is extremely, how angry would you feel in this moment?**

(not at all) 1 2 3 4 5 6 7 (extremely)

**Part 2. High Benefit Condition**

The story continues here...

While you’re picking up your fallen bags, you hear the guy talking on the phone with a friend.

“Alexandre, grab it if you’re there! Alexandre? Good, you’re still there. I just saw the number on the lottery ticket; I won. I think Pat threw it in the trash with the rest of the stuff. You need to get it out of the trash before the garbage truck comes at 5:00 PM. Is the trash still there?” He pauses for a moment. “Great, thanks a lot.”

He puts the phone down and whispers to himself, “I can’t believe I almost lost a R$5.000,00 ticket.”

**Compared to your previous answer, how much anger would you feel now?**

-3 (much less anger) -2 -1 0 (the same anger) 1 2 +3 (much more anger)

How happy would you feel now?

(not at all) 1 2 3 4 5 6 7 (very strongly)

How surprised would you feel now?

(not at all) 1 2 3 4 5 6 7 (very strongly)

How sad would you feel now?

(not at all) 1 2 3 4 5 6 7 (very strongly)

How scared would you feel now?

(not at all) 1 2 3 4 5 6 7 (very strongly)

How envious would you feel now?

(not at all) 1 2 3 4 5 6 7 (very strongly)

How embarrassed would you feel now?

(not at all) 1 2 3 4 5 6 7 (very strongly)

How compassionate would you feel now?

(not at all) 1 2 3 4 5 6 7 (very strongly)

**Part 2. Low Benefit Condition**

The story continues here...

While you’re picking up your fallen bags, you hear the guy talking on the phone with a friend.

“Alexandre, grab it if you’re there! Alexandre? Good, you’re still there. I just saw the number on the lottery ticket; I won. I think Pat threw it in the trash with the rest of the stuff. You need to get it out of the trash before the garbage truck comes at 5:00 PM. Is the trash still there?” He pauses for a moment. “Great, thanks a lot.”

He puts the phone down and whispers to himself, “I can’t believe I almost lost a R$5,00 ticket.”

**Compared to your previous answer, how much anger would you feel now?**

-3 (much less anger) -2 -1 0 (the same anger) 1 2 +3 (much more anger)

How happy would you feel now?

(not at all) 1 2 3 4 5 6 7 (very strongly)

How surprised would you feel now?

(not at all) 1 2 3 4 5 6 7 (very strongly)

How sad would you feel now?

(not at all) 1 2 3 4 5 6 7 (very strongly)

How scared would you feel now?

(not at all) 1 2 3 4 5 6 7 (very strongly)

How envious would you feel now?

(not at all) 1 2 3 4 5 6 7 (very strongly)

How embarrassed would you feel now?

(not at all) 1 2 3 4 5 6 7 (very strongly)

How compassionate would you feel now?

(not at all) 1 2 3 4 5 6 7 (very strongly)

**Lunch vignette**

**Part 1.**

Read the following description carefully as if you were really in this situation:

You and a friend sign up for a biology class at university that requires you to go out with the group and observe wildlife in the forest. The Saturday trips take place between 10:00 AM and 3:00 PM, so students bring their lunches, which are kept on the bus to avoid attracting wildlife.

You know the other students on the trip, but almost everyone stays with their lab partners. Everyone has already chosen their lab partners, so you and your friend worked together. One student, João, is a bit playful and occasionally tells one or two offensive jokes. One day, when everyone is back on the bus, the teaching assistant opens the trash can where the lunches are stored and you see that one of the lunches is marked with the phrase: "Finally, meet something as smart as you." The assistant notices that the bag is moving, and when they open it, a ten-centimeter slug crawls out. Everyone bursts out laughing. One of your friends congratulates João on the joke. "Nice," he says. "Thanks," João replies.

It’s your lunch. Everyone watches as you heat it up and throw the slug aside.

Please answer the following question before continuing with the story:

**On a scale of 1 to 7, where 1 is not at all and 7 is extremely, how angry would you feel in this moment?**

(not at all) 1 2 3 4 5 6 7 (extremely)

**Part 2. Specific Victim Condition**

The story continues here...

While people continue to laugh at you, one of the other students whispers to João:

"Did you know whose lunch it was?"

João answers: "Yes."

**Compared to your previous answer, how much anger would you feel now?**

-3 (much less anger) -2 -1 0 (the same anger) 1 2 +3 (much more anger)

How happy would you feel now?

(not at all) 1 2 3 4 5 6 7 (very strongly)

How surprised would you feel now?

(not at all) 1 2 3 4 5 6 7 (very strongly)

How sad would you feel now?

(not at all) 1 2 3 4 5 6 7 (very strongly)

How scared would you feel now?

(not at all) 1 2 3 4 5 6 7 (very strongly)

How envious would you feel now?

(not at all) 1 2 3 4 5 6 7 (very strongly)

How embarrassed would you feel now?

(not at all) 1 2 3 4 5 6 7 (very strongly)

How compassionate would you feel now?

(not at all) 1 2 3 4 5 6 7 (very strongly)

**Part 2. Random Victim Condition**

The story continues here...

While people continue to laugh at you, one of the other students whispers to João:

"Did you know whose lunch it was?"

João answers: "No."

**Compared to your previous answer, how much anger would you feel now?**

-3 (much less anger) -2 -1 0 (the same anger) 1 2 +3 (much more anger)

How happy would you feel now?

(not at all) 1 2 3 4 5 6 7 (very strongly)

How surprised would you feel now?

(not at all) 1 2 3 4 5 6 7 (very strongly)

How sad would you feel now?

(not at all) 1 2 3 4 5 6 7 (very strongly)

How scared would you feel now?

(not at all) 1 2 3 4 5 6 7 (very strongly)

How envious would you feel now?

(not at all) 1 2 3 4 5 6 7 (very strongly)

How embarrassed would you feel now?

(not at all) 1 2 3 4 5 6 7 (very strongly)

How compassionate would you feel now?

(not at all) 1 2 3 4 5 6 7 (very strongly)

**Arguments/Reactions vignettes**

**Argument Scenario**

We are interested in how people argue.

Below, we describe some situations where someone is angry at you because of something you did.

We will then list a series of statements that you could use in your argument. Your task is to decide which statements strengthen your position and which weaken it.

For example, if a professor accuses you of cheating on a test, you could defend yourself by saying, "My grades were lower than rock bottom." This would weaken your defense.

Many conflicts are described in the following pages. Read them carefully. Below each one is a series of statements with a blank space before them. Some of these statements may contradict each other.

Evaluate each statement as if it were the only argument you are using in your favor.

**Use this scale:**

**-3 -2 -1 0 +1 +2 +3**

**Definitely does not help me Definitely helps me**

If a statement strengthens your argument, give it a positive value (For example, if it helps you a lot, give it a +3; if it only helps you a little, give it a +1).

Assign a zero to the statement if it neither helps nor hurts you.

Assign negative numbers to statements that definitely harm you (For example, if it harms you a lot, give it a -3; if it only harms you a little, give it a -1).

There are no tricks. We are interested in your immediate reaction.

**Imagine you ruined a friend’s shirt. They can no longer use it and are very upset with you. You want to argue that what you did was not so bad. Assume that they will believe the argument you use.**

**How much would each statement strengthen or weaken your position?**

1. He bought the shirt at a thrift store for five reais.

-3 -2 -1 0 +1 +2 +3

Definitely does not help me Definitely helps me

2. His grandmother gave him the shirt before she died.

-3 -2 -1 0 +1 +2 +3

Definitely does not help me Definitely helps me

3. You ruined his shirt by using it to clean your bathtub.

-3 -2 -1 0 +1 +2 +3

Definitely does not help me Definitely helps me

4. You ruined his shirt to stop the bleeding from a head wound after a car accident.

-3 -2 -1 0 +1 +2 +3

Definitely does not help me Definitely helps me

5. You didn’t know the shirt was his when you ruined it.

-3 -2 -1 0 +1 +2 +3

Definitely does not help me Definitely helps me

6. You knew the shirt was his when you ruined it.

-3 -2 -1 0 +1 +2 +3

Definitely does not help me Definitely helps me

**Reaction Scenario**

We are interested in how people react to arguments.

Below, we describe some situations where you are angry about something others have done to you. They don't seem to think what they did was very bad, but you do (at least at first). Then, we will list a series of facts that may be related to your anger.

It’s up to you to decide which facts would make you angrier and which would make you less angry. Some of these facts may contradict each other.

Evaluate each fact as if it were the only fact you are considering.

**Use this scale:**

**-3 -2 -1 0 +1 +2 +3**

**Definitely makes me less angry Definitely makes me more angry**

If a fact strengthens your anger, give it a positive value (For example, if it makes you much angrier, give it a +3; if it only makes you a little angrier, give it a +1).

Assign a zero to the fact if it does not affect your anger.

Assign negative numbers to facts that definitely make you less angry (For example, if it makes you much less angry, give it a -3; if it only makes you a little less angry, give it a -1).

There are no tricks. We are interested in your immediate reaction.

**Imagine a friend ruined one of your shirts. Your shirt is completely ruined, and you are very upset about it. Your friend doesn't think what they did was so bad.**

**How would each fact strengthen or weaken your anger in each situation?**

1. You bought the shirt at a thrift store for five reais.

-3 -2 -1 0 +1 +2 +3

Definitely makes me less angry Definitely makes me more angry

2. Your grandmother gave you the shirt before she died.

-3 -2 -1 0 +1 +2 +3

Definitely makes me less angry Definitely makes me more angry

3. He ruined your shirt by using it to clean his bathtub.

-3 -2 -1 0 +1 +2 +3

Definitely makes me less angry Definitely makes me more angry

4. He ruined your shirt to stop the bleeding from a head wound after a car accident.

-3 -2 -1 0 +1 +2 +3

Definitely makes me less angry Definitely makes me more angry

5. He didn’t know the shirt was yours when he ruined it.

-3 -2 -1 0 +1 +2 +3

Definitely makes me less angry Definitely makes me more angry

6. He knew the shirt was yours when he ruined it.

-3 -2 -1 0 +1 +2 +3

Definitely makes me less angry Definitely makes me more angry

**References**

Brown, T. (2015). *Confirmatory Factor Analysis for Applied Research (2nd Ed)*. Guilford Press.

Valentini, F., & Damásio, B., F. (2016). Variância Média Extraída e Confiabilidade Composta: Indicadores de Precisão. *Psicologia: Teoria e Pesquisa*, *32*(2).<https://doi.org/10.1590/0102-3772e322225>

DiStefano, C., Morgan, G. B. (2014). A Comparison of Diagonal Weighted Least Squares Robust Estimation Techniques for Ordinal Data. *Structural Equation Modeling, 21*(3), 425-438.<https://doi.org/10.1080/10705511.2014.915373>

Li, C. H. (2016). Confirmatory factor *analysis* with ordinal data: Comparing robust maximum likelihood and diagonally weighted least squares. *Behavioral Research Methods, 48*(3), 936-49.<https://doi.org/10.3758/s13428-015-0619-7>

Raykov, T. (1997). Estimation of composite reliability for congeneric measures. *Applied Psychological Measurement, 21*(2), 173-184.<https://doi.org/10.1177/01466216970212006>

Sznycer, D., Al-Shawaf, L., Bereby-Meyer, Y., Curry, O. S., De Smet, D., Ermer, E., Kim, S., Kim, S., Li, N. P., Lopez Seal, M. F., McClung, J., O, J., Ohtsubo, Y., Quillien, T., Schaub, M., Sell, A., van Leeuwen, F., Cosmides, L., & Tooby, J. (2017). Cross-cultural regularities in the cognitive architecture of pride. *Proceedings of the National Academy of Sciences*, *114*(8), 1874–1879. <https://doi.org/10.1073/pnas.1614389114>

Sznycer, D., Tooby, J., Cosmides, L., Porat, R., Shalvi, S., & Halperin, E. (2016). Shame closely tracks the threat of devaluation by others, even across cultures. *Proceedings of the National Academy of Sciences*, *113*(10), 2625–2630. https://doi.org/10.1073/pnas.1514699113
